# Establishment of the Namibian National Public Health Institute: laboratory systems strengthening

**DOI:** 10.1093/eurpub/ckac131.033

**Published:** 2022-10-25

**Authors:** C Thieleke-Matos, N Endjala, E Nepolo, C Winter, V Nowaseb, M Mataranyika, P Ochurus, A Jansen, S Weiss, H Ellerbrok

**Affiliations:** 1 Robert Koch Institute, Berlin, Germany; 2 Namibia Institute of Pathology, Windhoek, Namibia; 3 University of Namibia, Windhoek, Namibia; 4 Ministry of Health and Social Sciences, Windhoek, Namibia

## Abstract

In 2020, the Namibian Ministry of Health and Social Services (MoHSS) and the Robert Koch Institute (RKI) started a twinning project with the long-term goal of establishing a Namibia Institute of Public Health (NIPH). A fundamental pillar of an NIPH is a fully operational Public Health laboratory system. Due to the COVID-19 pandemic, the need for strengthening the existing Namibian Laboratory system became eminent. Following the Intra-Action Review (IAR) of the COVID-19 response in Namibia in 2020, three regional diagnostic laboratories, at points of entry, were assessed. The major issues identified were long delays between sampling of both suspected cases and COVID-19 patients and receiving test results due to extended sample transport times to the central laboratory in Windhoek and the overload of the central capacities due to overwhelming numbers of samples during peak times. This led to the establishment of three SARS-CoV-2 PCR diagnostic laboratories through procurement and installation of equipment, provision of consumables/reagents, and on-site training of laboratory technicians with continued virtual technical support. Consequently, an important reduction of the diagnosis turnaround time was achieved. Of great value was the technical support given by the staff at the central laboratory during the trainings allowing for immediate validation of the newly established laboratories and to strengthen the communication between regional laboratories and the central laboratory. The Namibian molecular diagnostic capacities have increased in important regions in Namibia and will provide data to support the health policies of the future NIPH. New diagnostic protocols will be developed to foster the sustainability of the established laboratories and could support the implementation of genomic surveillance capacities. Finally, stronger relationships were built through these joint activities, which will support and the next steps of the establishment of the NIPH.

**Key messages:**

• Supporting and Strengthening the Namibian Public Health Laboratory system.

• Long-term goal of establishing a Namibia Institute of Public Health (NIPH).

